# Remarks on the Pathology of Congenital Hydrocele

**Published:** 1897-07

**Authors:** Charles Greene Cumston

**Affiliations:** Assistant professor of surgical pathology. Faculty of Medicine, Tufts College, Boston; corresponding member of the Association of Genito-urinary Surgeons of France, etc.


### REMARKS ON THE PATHOLOGY OF CONGENITAL HYDROCELE.

By CHARLES GREENE CUMSTON, B. M. S., M. D.,

Assistant professor of surgical pathology, Faculty of Medicine, Tufts College, Boston; corresponding member of the Association of Genito-urinary Surgeons of France, etc.

THE word hydrocele in its rigorous etymological sense means any tumor which contains a serous liquid, and is consequently the synonym of hydropsy. The older surgeons understood it in this way, and Sabatier wrote on it in 1796 in the following terms: "The name hydrocele is given to aquous tumors of the scrotum. These tumors are of two kinds: either the water is contained in the fatty tissue or else it is collected in one pocket. In the first case we have hydrocele by infiltration while in the second case we have a collected hydrocele."

Since this time, surgeons have used the word hydrocele in a very restricted way, and are in the habit of using it only to indicate an accumulation of serous liquid in the vaginal tunic of the testicle. But besides this simple vaginal hydrocele, we must consider a second variety. We all know that in the greater number of cases, the vagino-peritoneal canal which is patent during intrauterine life is obliterated at birth, but it is nevertheless not very infrequent to meet with a patient in which imperfect obliteration or even no obliteration whatever is present. Every time that a nonobliterated or imperfectly obliterated canal exists, a collection of serous fluid may manifest itself by various clinical symptoms, and then we have a congenital hydrocele. The adjective, congenital, is very proper, although some writers think it is not.

It is perhaps in this sense that this disease does not necessarily appear immediately after birth, but on the contrary is often met with in the adult and even in the aged. But as the form that it affects is intimately united to a vice of conformation of the enveloping tissues of the testicle, I cannot see any reason why it should not be considered as congenital in origin.

The definition that I would give for congenital hydrocele is *a collection of a serous liquid in the vagino-peritoneal canal which is incompletely or not at all obliterated.*

I am perfectly well aware that some of the modern authorities are reserved regarding the term of congenital hydrocele when applied to a sac freely communicating with the peritoneal cavity. But I do not see any reason for this, nor do I understand why they should attribute other theories to those serous cysts which are sometimes found on the spermatic cord. I think with the great majority of writers that these cysts are due to the presence in the cord of nonobliterated parts of the vagino-peritoneal canal. These aberrant diverticuli are in the beginning empty, but under some influence which sets up inflammation of their walls, they fill up with a serous liquid and thus form the cysts in question.

Congenital hydrocele is far from presenting the same anatomical type in every case, and there are numerous varieties which are easily understood if we will consider anatomy and embryology. And in the first place we will divide the various kinds of hydrocele in two large classes. The first class comprises communicating hydrocele, that is to say those which are made up by the vagino-peritoneal canal and thus remain in communication with the peritoneal cavity.

The second class comprises hydrocele formed by some part of the vagino-peritoneal canal, but which has lost all communication with the peritoneal cavity. This hydrocele may be called encysted. Of course it is understood that I do not include ordinary hydrocele of the vaginal tunic, which is nothing more than an encysted hydrocele in a normal cavity.

Let us now consider the first class or communicating hydrocele.

The first variety is formed by a hydrocele which has its homologue in vagino-testicular hernia, and I would call this variety peritoneo-testicular hydrocele. This is the most frequent type and is the result of a complete nonobliteration of the vagino-peritoneal canal.

In the case in which the fourth stricture exists, which as we know is not always present in the vagino-peritoneal canal, we have to deal with a peritoneo-funiculo-testicular hydrocele and this corresponds to the type of funiculo-testicular hernia.

If nonobliteration of the vagino-peritoneal canal only extends to the entire peritesticular vaginal serous membrane, we have the type of peritoneo-funicular hydrocele which corresponds to funiculo-vaginal hernia.

Now let us suppose that the obliteration of the vagino-peritoneal canal stops at the inguinal ampulla, we then have a peritoneo-inguinal hydrocele which is similar to the interstitial inguinal hernia.

On the other hand, if the obliteration of the vagino-peritoneal canal extends up to the properitoneal ampulla we have the peritoneo-properitoneal hydrocele which corresponds to the properitoneal inguinal hernia.

These five varieties of hydrocele are not met [illegible] quency. The first two types are surely the mo[illegible] to the others they are probably very much more [illegible] what I would like to demonstrate is the striking si[illegible] congenital hydrocele and congenital hernia.

We all know that hernia is frequently co[illegible] genital hydrocele, from the fact of the persiste[illegible] peritoneal canal which forms an open passage th[illegible] intestine or mesentery pass. Epiplocele and entero[illegible] only accidents which may render the contents of a con[illegible] cele most varied. Sometimes we will find that there [illegible] of the testicle, as well as some diverticuli, the natur[illegible] still a very disputed question.

Considering now the question of encysted hydrocele, it may be said that they can also be very properly called funicular hydrocele, because they are always to be found on some part of the spermatic cord, corresponding to a nonobliterated portion of the vagino-peritoneal canal.

The first variety of this type of hydrocele comprises the hydrocele "en bissac" of Dupuytren and should be attributed to the persistence of a more or less considerable portion of the vagino-peritoneal canal which is only obliterated at its upper part and continues below with the vaginal tunic of the testicle.

The second variety of encysted hydrocele is the encysted hydrocele of the cord properly speaking. This hydrocele represents the remains of an incompletely obliterated vagino-peritoneal canal and may be seated quite high up in the interior of the inguinal canal, but generally it is near the external ring of the great oblique muscle through which it projects.

Duplay has mentioned a third variety of encysted hydrocele occurring in the vaginal serous membrane of the testicle. This variety is to be considered rather as an aberrant type of ordinary hydrocele, because it has nothing to do with the nonobliteration of the vagino-peritoneal canal.

The pathogenesis of congenital hydrocele is one of the most obscure questions of modern surgery and many theories have been admitted to explain the origin of the serous liquid which they contain. The production of hydrocele on account of the poor general health of the mother or the child, can be admitted, because we know very well that certain diseases, such as those of the nervous system, rheumatism and syphilis, act on the system of the child as
[illegible] mother. But as yet nobody has found that I
[illegible] completely localised affection which may be
[illegible] paternal or maternal antecedents; or if such
[illegible] be brought up, they are without doubt a simple
[illegible] places the origin of the pathological liquid
[illegible] the serous membrane lining the vagino-
[illegible] and is the consequence of a molecular process
[illegible] sought for in the development of the fetus

[illegible] the testicle has come down, that is to say usually

[illegible] third month of intrauterine life, sometimes later, a

[illegible] phenomena take place in the vagino-peritoneal canal

[illegible] for a result the closing of the cavity, which then will present all the characters of a serous cavity and which will be retained after the child is born.

The obliteration is the result of the phenomena that Hunter long ago designated under the name of adhesive inflammation. This process does not follow an irritation produced by the testicle at the time when it enters the scrotum: it is constant, because it is intimately united to the nutritive activity and evolution which is the elementary principle of the development of man from the first embryonic period up to old age. It is only, consequently, the continuation of this inflammatory process itself, in which, according to German writers, human life appears to have its origin.

If we examine the manner of obliteration of the sac we will first notice a falling off of the epithelium which is the first condition for adhesions to occur between two serous surfaces. Soon we will see a neomembrane appear made up of young connective tissue elements and supplied by a very large number of capillary vessels. An anastomosis is not long in taking place between these newly formed vessels and thus an adhesion is formed. Nevertheless in order that the inflammatory process ends in an obliteration, it is not sufficient that there be a desquamation of the serous membrane and the formation of neomembranes, but it is necessary that both the walls of the cavity be in contact in order that the adhesion can occur. These conditions, unless they be exceptional pathological circumstances, are realized in the vagino-peritoneal canal. But low down at the place where this canal increases in size, in order to form a cavity, the two surfaces are no longer in such favorable conditions for the formation of adhesions.

By its many movements in the scrotum, the testicle prevents the visceral layer which covers it from making any intimate adhesions with the parietal serous membrane and, consequently, the processes which in the canal end in obliteration may also take place here, but the two walls not being usually in contact cannot become adherent and an excremental product is formed consisting of an albumino fibrinous fluid. This is so true that by examination of the liquid collection a simple quantity of endosmo-exosmotic serosity is not only found, but an exudation of principals which are due to inflammation of the serous membrane are present as well. Lannelongue has always found quite large quantities of fibrine, and if it is true that sometimes this substance is also found in hydrocele in the adult, as has been demonstrated by Mehol, it is well to add that when there is any, it is always in exceedingly small quantity.

Thickening of the vaginal tunic that has always been found by Lannelongue testifies to an inflammatory condition. The skin, instead of preserving its normal color, is often red and erythematous, which is always considered as due to friction during labor and to the irritation resulting from the constant contact with the urine; but this inflammatory condition may also be a distant phenomena of the same conditions of development, because I have sometimes remarked that children of fourteen or sixteen months of age, and who had hydrocele from birth, and were kept in the very best of hygienic conditions, presented this condition of the scrotum.

The inflammation is never sufficiently acute to end in a phlogosis, which histologically is found to be hemorrhagic, red and white blood corpuscles being present. The processes take place slowly and the exudation continues to be produced in the vaginal membrane up to the time that an unequal activity between the organism and the collected liquid occurs, when absorption takes place.

But usually a collection thus produced becomes in itself a permanent cause of irritation and keeps up the inflammation. Consequently when this pathological process has lasted for a certain time, there is a change in the structure of the tissue and absorption can no longer take place unless an appropriate treatment is adopted. The membrane is thick and hard and is no longer capable of receiving the liquid which ought to be absorbed. Then when the sac is emptied the liquid will form again, and after each puncture the same phenomena will occur; thus a cure cannot be brought about, because the lack of tonicity of the vessels continues to allow the passage of serosity through their walls and the inflammation is not sufficiently intense for the formation of neomembranes sufficiently resistant and durable; whereas if the circulation was more active, the thinness of the walls would allow the liquid to be absorbed. The membranes will disappear of their own accord when the walls which support them have become normal.

After these facts it seems probable that congenital hydrocele of the vaginal tunic is simply a phenomena of development. Now the question arises, Why does not a liquid collection take place in every case? To answer this question we must remember that the constitution and temperament have certainly a relationship to the quantity and quality of the liquid and certainly play a large part. In a strong child of a sanguinous temperament the collection will occur more rapidly than in one with a lymphatic temperament, but nevertheless absorption will be just as complete in either. I have noticed that these liquid collections which occur with rapidity undergo a rapid absorption.

This theory of Lannelongue which in the first place appears very acceptable does not, however, fully convince me that this is the real truth of the matter, but I am far from denying that inflammatory phenomena cannot occur in the vagino-peritoneal canal, but I do not think they can take place unless some accident arises which is the starting point.

Several authorities have considered the origin of hydrocele as due to the compression of the vessels which enter into the vaginal tunic or to the spermatic vessels. They have also incriminated all sorts of pressure to which a child is exposed during labor, especially if the latter is a difficult one, and during the early part of its existence when the cries and the efforts of the child push the viscera against the spermatic vessels and thus compress them. These same vessels may also be pressed upon at this age in the inguinal canal, especially at its orifices.

For other authorities, a large share should be given to the difficulties of labor and especially breech presentations in the pathogenesis of congenital hydrocele. It happens that in these cases the parts near the scrotum and the scrotum itself are compressed by the uterine contractions and the serous membrane of the testicle becomes the seat of an abundant afflux of blood.

Nelaton believed that friction of the scrotum, as well as irritation by contact with urine and feces, may explain the increase of the secretion in the vaginal tunic. This theory has also been adopted by several authorities, and Sejournet says that the conditions in which we observe the origin and the progress of the vaginal collection have in a certain measure given us a clue to the pathogenesis of hydrocele in the newly-born. The children that the latter authority has had under his care and who presented a hydrocele, had all an erythema, sometimes localised at the genital organs, at others it was generalised over the buttocks, thighs, scrotum and penis. This eruption was red, shiny and hot and appeared to be the cause of the hydrocele. The cutaneous affection little by little extended to the canal of the urethra and from there reached the cord and the vaginal tunic, in which it produced a liquid collection. This pathological extension appeared to explain all, because the following steps were noted, viz.: genital erythema, urethritis, funiculitis, epididymitis and lastly vaginalitis, such were the successive steps taken by the inflammatory process.

What confirms Sejournet in his opinion is, in the first place, the existence of a urethritis which caused painful micturition, because the child cried out each time he passed his water, as well as tumefaction of the spermatic cord found in some few cases, thus indicating a certain degree of funiculitis, and sometimes also he found the epididymis enlarged and the testicle was also larger than its mate.

This manner of progression of the inflammation is similar to that of erysipelas extending into the depth of the genital organs, and that it was a real ascending infection cannot be held in doubt, because in the cases he reports there was always a coincidence of hydrocele, or, better, orchi-vaginalitis, with erythema of the genital organs.

Some authorities, principally German surgeons, consider ectopia of the testicle as a cause of congenital hydrocele. Ectopia is not infrequent, as is shown by the statistics of Trélat, Holmes, LeDentu and others. According to this hypothesis the testicle can provoke a serous collection in two different ways: either by acting like a foreign body, for example as in inguinal ectopia, where it produces an inflammation of the serous membrane capable of producing a liquid; or by sudden movements or a shock, the gland itself becomes irritated and produces an inflammation of the vaginal serous membrane. Wechselmann mentions a case in which the left testicle was not in the scrotum at birth; a few days afterward it had descended into its normal place, but a liquid collection could be felt in the vaginal tunic. Chassaignac mentions the presence of hydrocele following a tardy descent of the testicles, while Labat out of sixteen cases only met with this condition of affairs once.

Marimont called attention in 1874 to cysts of the epididymis. The pathogenesis of these cysts has been studied thoroughly by Poirier, who has been able to demonstrate that they develop in the vaginal serous membrane. Consequently it may be asked if the presence of these small cysts may not have an influence on the production of liquid in the vaginal serous membrane and this may be the case, because by their presence they play the part of a foreign body and thus are the cause of irritation. Let me remark that these cysts which are well known in the adult and in the aged are not well known in children.

According to Guersant the production of congenital hydrocele is to be easily understood. During intrauterine life at the time when the testicles go down into the scrotum it is easy to admit that some of the serous liquid contained in the peritoneal cavity accompanies these organs and enters into the sac formed by the vaginal tunic along with them. The presence of this liquid may be the means of preventing the obliteration between the two serous membranes.

We now come to a new theory which attributes the origin of the liquid contained in hydrocele to the peritoneal cavity and thus supposes that there was a preëxisting fluid in the peritoneum.

The experiments of Richet and Delbet have perfectly demonstrated that the peritoneal cavity is absolutely free from any liquid or moisture when in the normal condition. It is consequently necessary to admit that there has been some pathological process to which is due the production of the peritoneal liquid and Verneuil understood this perfectly well, for he says that the nature and the origin of congenital hydrocele demands that the anatomical conditions, which are a communication between the vaginal sac and the peritoneal cavity and pathological changes of the peritoneum, that is to say, a hypersecretion of this membrane sufficiently intense to be forced through the ring, must be present.

Now, what are these pathological conditions? The possibility of an essential peritoneal hydropsy which is idiopathic, occurring spontaneously without any pathological cause and leaving no traces of inflammation after it has been put forward as an explanation. But those writers who have admitted this supposition have only based it on incomplete observation.

Then came the theory of inflammation of the peritoneum, due to the abnormal presence of the testicle in the abdomen. According to the partisans of this theory, the testicle produces a slight irritation of the peritoneum which is sufficient to produce the pathological exudation. Richter, after many careful researches, only found vaginal hydrocele in one case out of many in which the testicle had not entered the scrotum.

According to Faure the peritoneum does not need to be inflamed in order to secrete an exudate. It is sufficient to have an increase of pressure in the subperitoneal vessels and particularly in the portal system. It is for this writer rather more of a pathological condition of the peritoneum, which without any local symptom is powerfully felt throughout the entire economy. In order to uphold his theory he brings forward the abundance of the collection which, in order to arrive at the orifice of the vagino-peritoneal canal, must necessarily fill the entire pelvis and also the spontaneous disappearance of the liquid or when this occurs after a bandage has been worn.

This last theory has been vigorously attacked by Blum, who asks how a bandage may influence the condition if the peritoneum is thus altered, and what becomes of the liquid secreted when the vagino-peritoneal canal becomes obliterated? If this were the case, would the liquid not continue to increase within the abdomen and thus produce an ascitis which would be remarked by the clinical signs to which it would give rise? Or, what would be this special pathological condition of the peritoneum which after producing so large a collection would disappear entirely of itself?

Now, in the first place it must be remembered that the cure of a congenital hydrocele following an obliteration of the vagino-peritoneal canal is no myth and Curling has seen a cure result after a bandage has been worn. The radical cures obtained lately after obliteration of the canal also pleads in favor of this opinion and the cases reported by Arnison, Southam and Phocas all go to prove this.

Now, as to the existence of this ascitis that according to Blum should be found if the liquid has really a peritoneal origin, and it certainly occurs in a good many cases, Phocas says that the peritoneal origin of these collections may be demonstrated clinically. In three cases he was able to demonstrate this without a doubt by finding not only dulness on percussion, but a sensation of liquid which was perceptible in the subumbilical region of the abdomen. In the first case the latter symptom was only felt two months after a successful operation for the hydrocele but before the operation there was a subumbilical dulness. The second case was a child of six years, who presented a hydrocele, on the right side in a sac containing a congenital hernia which had been treated by bandages. The supra-umbilical veins were quite large and symptoms of ascitis were very distinct.

These facts led Phocas to carefully explore the abdomen in cases of congenital hydrocele and nearly always he has been able to find a slight dulness in the hypogastric region, which he attributes to a certain quantity of liquid contained in the abdomen. I have found an ascitis, with congenital right-sided hydrocele, in a child with marked symptoms of hereditary syphilis. Phocas further says that in a certain number of other cases he was able to discover the nature of the ascitis and to demonstrate the real cause of hydrocele and mentions the cases reported by Lorain and Letulle, in which purulent peritonitis gave rise to a congenital hydrocele. But in the cases which Phocas observed it was tuberculosis of the peritoneum which showed its presence by a congenital hydrocele.

In most of the cases which he relates and especially the first, the tuberculous nature of the hydrocele appears to me perfectly correct; the only question is to know if the tuberculosis commenced in the peritoneum and from there extended down the vagino-peritoneal canal or whether the opposite occurred.

If congenital hydrocele that is observed in these cases was only a symptom of an ascitis, it would hardly be worth while mentioning the fact and a hydrocele would then be very similar to the liquid of an omphalocele, which is frequently met with in cases of ascitis. But here it is not the ascitis which first calls the attention of the surgeon, but it is the hydrocele. The whole clinical picture is dominated by the presence of a classical reduceable hydrocele occurring in a child. The patient's parents only consider the local trouble and it is only for this that they seek advice.

The relationship which unites these coinciding affections—namely, hydrocele and ascitis,—is not easy to determine. Two hypotheses may be admitted.

In the first, the following hypothesis may be offered to explain the succession of the pathological phenomena. A certain quantity of liquid arises in the peritoneum and this liquid being in small quantity gravitates to the inclined portions of the serous membrane. Then, at a certain time, it collects in the vagino-peritoneal canal, which by a congenital defect has remained unobliterated.

Consequently, it is on account of this congenital defect as well as mechanically that a hydrocele is formed after a tuberculous ascitis, as it could be produced by any kind of ascitis occurring in a subject in which nonobliteration of the canal existed. It would be more simple to compare a hydrocele of this type to a liquid omphalocele which so often accompanies ascitic collections.

In the second hypothesis we may consider the condition in a different manner. In a child with a predisposition to tuberculosis, an unobliterated vagino-peritoneal canal may become the seat of a local tuberculosis produced by a traumatism, general disease or some other cause. This tuberculosis produces a serous collection in the canal, but on account of the large communication of the former with the peritoneal cavity, the infection extends to the peritoneum. Thus in this hypothesis the vagino-peritoneal canal plays the most important part.

The majority of reported cases would lead me to adopt the latter as most probable, because peritonitis in 75 per cent. of the cases reported was only a secondary affair in the symptomatological picture. The children were brought for a hydrocele, and the peritonitis being latent, produced few functional troubles for a long time.

Jonnesco has collected nine published cases of tuberculosis in hernia, but in the majority of cases reported by Phocas the congenital hydrocele was not tuberculous.

There is a particular form of congenital hydrocele which may be the first symptom of a tuberculosis of the peritoneum, but the presence of this form of hydrocele still remains to be studied.

Now, is this variety of hydrocele frequent? It probably is, but reported cases are not conclusive. Perhaps some have been reported, but they are not as yet generally known and for several reasons. In the first place it may happen that the severe symptoms of visceral tuberculosis takes up the entire attention of the physician in charge, who neglects finding the hydrocele and ascitis, especially if, as it often happens, the latter is difficult to discover, being only detected by percussion. On the other hand, there are many examples of beginning tuberculous peritonitis which get well after having produced a slight exudation in the peritoneal cavity.

A few words now on the diagnosis of tuberculous hydrocele in children.

The tumor of the scrotum is the first, because it is usually this that brings the patient to the surgeon. In the majority of cases the tumor is on the right side and the presence of liquid is quickly made out by transparency and fluctuation. As we are always dealing with a communicating hydrocele the fluid may be pushed into the peritoneum, but not always with the same ease in every case. Sometimes if the patient is made to lie down the liquid will disappear, but at others we may be obliged to perform taxis in order to obtain the same result. In some cases it may be necessary to keep the patient lying down for some time before reduction can be accomplished.

Reduction is usually made without any noise unless there is a hernia complicating the hydrocele, in which case the characteristic sound as the intestine passes into the peritoneal cavity will be heard. As to the size of the sac it varies according to the case and also with the time of the day: it increases under the influence of coughing or an effort and it may acquire such a tension that fluctuation may be wanting. Not infrequently the cord and epididymis are increased in size and are sensitive to pressure.

Sometimes there may be more or less considerable swelling of the abdomen and a complementary supra-umbilical veinous circulation and then one may be able to elicit the presence of liquid. But usually these phenomena are not very marked and they must be made out by percussion of the abdomen and a zone of dulness will be found extending above the pubis and over the iliac fossa, whose principal character is to become displaced when the patient changes his position. It is also well to ascertain if there is any pain on pressure in the right iliac fossa.

The general condition of the patient will be usually that found in tuberculosis, but this may not always be the case. Some patients will present some signs of a visceral tuberculosis.

The diagnosis of tuberculous hydrocele should consider the presence of a coinciding ascitis and secondly, the nature of this ascitis.

A hydrocele will generally be easily made out by the ordinary signs. Nevertheless a congenital hernia may coëxist, in which case the diagnosis is more delicate. As to the ascitis it is often difficult to make out, but if the patient be carefully examined several times a day and care is taken to empty his bladder, the making of the diagnosis will be rendered easier.

And lastly, in order to find out the nature of the hydrocele and the coëxisting ascitis, we should take into account both hereditary and personal antecedents of the patient, the general condition, the condition of the viscera and in particular the lungs. Every time that it is possible we should examine the liquid taken from the hydrocele by an aspirating needle, in order to make cultures and experimental inoculations in animals. In closing this paper, I would say that beside the tuberculous congenital hydrocele, which has been described by Phocas, there are also hydroceles which are symptomatic of ascites having a different nature, as of syphilis for example.

871 Beacon Street.

